# Draft Genome Sequences of Three Fusarium oxysporum f. sp. *niveum* Isolates Used in Designing Markers for Race Differentiation

**DOI:** 10.1128/MRA.01004-20

**Published:** 2020-10-15

**Authors:** Owen Hudson, Dylan Hudson, Pingsheng Ji, M. Emran Ali

**Affiliations:** a Department of Plant Pathology, Coastal Plain Experiment Station, University of Georgia, Tifton, Georgia, USA; b Independent researcher, Boulder, Colorado, USA; Broad Institute

## Abstract

Here, we report the draft genome sequences of three Fusarium oxysporum f. sp. *niveum* isolates that were used to design markers for molecular race differentiation. The isolates were collected from watermelon fields in Georgia (USA) and were determined to be different races of *F. oxysporum* f. sp. *niveum* using a traditional bioassay.

## ANNOUNCEMENT

*Fusarium* wilt in watermelon is caused by the fungal pathogen Fusarium oxysporum f. sp. *niveum*, which is one of the most impactful pathogens for watermelon production worldwide (https://edis.ifas.ufl.edu/PP352) (1–3). Three isolates of *F. oxysporum* f. sp. *niveum* were obtained from infected watermelon plants (Citrullus lanatus) with typical symptoms of *Fusarium* wilt in commercial fields in Georgia. Traditional diagnostic methods are unable to identify the pathogen as *F. oxysporum* f. sp. *niveum* with 100% confidence, so molecular assays are required to confirm the identity of an isolate (4–6). Additionally, it is currently recognized that *F. oxysporum* f. sp. *niveum* has 4 races (R0, R1, R2, and R3) which require a bioassay for differentiation, as at the date of this writing, only race 2 may be distinguished molecularly (https://edis.ifas.ufl.edu/PP352) (1, 3, 7).

The three isolates that were chosen for sequencing were isolated from the lower stem (hypocotyl region) of diseased watermelon plants. Samples were surface-disinfested in 0.6% NaOCl, rinsed in sterile distilled water, and cultured on semiselective peptone pentachloronitrobenzene agar plates (8). Fungal cultures grown from the samples were identified based on the morphological characteristics, and single-spore isolates were grown on potato dextrose agar (PDA) plates and incubated at 25°C for 7 days. The races of the isolates were identified by inoculating differential watermelon plants and evaluating the disease development as reported previously (5, 7). Three isolates, representing three races of *F. oxysporum* f. sp. *niveum*, were grown on PDA for 10 days; next, 100 mg of mycelia was used for DNA extraction. DNA was extracted using the DNeasy plant minikit (Qiagen) and was concentrated and purified using Quantum Prep PCR Kleen Spin columns (Bio-Rad) according to the manufacturer’s instructions. PCR was performed on all isolates using FON-1/FON-2, primers specific to *F. oxysporum* f. sp. *niveum*, to confirm the identity of all *F. oxysporum* f. sp. *niveum* isolates, and the race 2-specific primer set FON*SIX*6F/R to confirm the race 2 isolate (9, 10). DNA was standardized at 200 ng/µL for each extraction and submitted to Novogene Co., Ltd. (Beijing, China) for whole-genome sequencing.

Libraries were prepped using an NEB Ultra II kit and sequenced using the paired-end strategy PE150 on an Illumina NovaSeq 6000 platform. The original optic data obtained by high-throughput sequencing were transformed into raw sequenced reads using Casava v.1.8 base calling and stored in FASTQ (fq) format. Quality control was performed using readfq v.10 (https://github.com/lh3/readfq) using default parameters, and low-quality sequences were removed (11, 12). Raw reads were assembled into scaffolds using SPAdes v.3.14.1 with the k-mer values 21, 33, 55, and 77 (13). Bowtie 2 v.2.4.1 was used to align the paired-end reads against the scaffolds produced by SPAdes, producing the SAM alignment files (14). SAMtools v.1.10 was used to convert the alignment files to BAM format and then sort and index the BAM files. Pilon v.1.23 (using - -frags mode) was used to polish the BAM files, yielding the final output of FASTA files (15, 16). Contigs shorter than 200 bp were removed from polished FASTA files using a novel Python script for contig filtration (17). The genome characteristics and accession numbers are given in Table 1.

**TABLE 1:** Genome assembly data and accession numbers for sequenced Fusarium oxysporum f. sp. *niveum* isolates

Isolate	Genome size (bp)	No. of contigs>50,000 bp	*N*_50_ (bp)	Avg coverage (×)	G+C content (%)	GenBank BioSample accession no.	GenBank BioProject accession no.	SRA accession no.
FONR1	61,207,430	245	154,443	28.65	48.79	SAMN15791673	PRJNA656528	SRR12492378
FONR2	54,074,873	217	161,737	22.92	47.53	SAMN15791674	PRJNA656528	SRR12492379
FONR3	55,220,015	220	158,709	22.38	47.54	SAMN15791675	PRJNA656528	SRR12492380

### Data availability.

All data for this whole-genome sequencing project were deposited under the GenBank BioProject accession number PRJNA656528. The raw reads of the genomic data for the isolates were deposited under the following SRA accession numbers: SRR12492378, SRR12492379, and SRR12492380. The accession numbers for each isolate are as follows: SAMN15791673, SAMN15791674, and SAMN15791675.
